# Supplementary material for: Breaking down barriers on PV trade will facilitate global carbon mitigation
Source: Nat Commun. 2021 Nov 24;12:6820. doi: 10.1038/s41467-021-26547-7 (PMC8613243; doi:10.1038/s41467-021-26547-7)
Supplement: Supplementary file 1 — Supplementary Information [file 41467_2021_26547_MOESM1_ESM.pdf]

Breaking down barriers on PV trade will facilitate global carbon mitigation

Supplementary Information

Mudan Wang [a, b], Xianqiang Mao [a, b, ✉], Youkai Xing[a, b], Jianhong Lu[a, b], Peng Song [c✉], Zhengyan Liu [b, d], Zhi Guo [a, b], Kevin Tu[e] and Eric Zusman [f,g]

[a] *School of Environment, Beijing Normal University, Xinjiekouwai Street No. 19, Beijing 100875, PR China*

[b] *Center for Global Environmental Policy, Beijing Normal University, Xinjiekouwai Street No. 19, Beijing 100875, PR China*

[c] *School of Public Affairs, Chongqing University, Shazheng Street No. 174, Chongqing 400044, PR China*

[d] *Institute of Spatial Planning and Regional Economy, China Academy of Macroeconomic Research, Beijing 100038, P. R. China*

[e] *Center on Global Energy Policy at Columbia University SIPA, 1255 Amsterdam Avenue, New York, NY 10027*

f Institute for Global Environmental Strategies, 2108-11 Kamiyamaguchi Hayama, Kanagawa, Japan

[g] *National Institute for Environmental Studies, 16-2 Onogawa, Tsukuba, Ibaraki, Japan*

✉ *maoxq@bnu.edu.cn; songpeng_ee@cqu.edu.cn*

1 Technical route of the present study

This study focuses on carbon emission reduction potential of global PV products trade and the trade barriers on solar cells and modules to carry out analysis. First, trade flow matrix (TFM) is constructed to describe the global PV products trade situation. Second, the carbon embodied in PV products trade are calculated. Third, the net carbon emission reduction potential of PV power generation is estimated and predicted up to 2060 with a bottom-up technology-based model, IMS. Finally, a computable partial equilibrium model, GSIM, is applied to simulate the impacts of trade barriers on PV product trade, and the gains and losses in carbon emissions reduction potential are calculated.

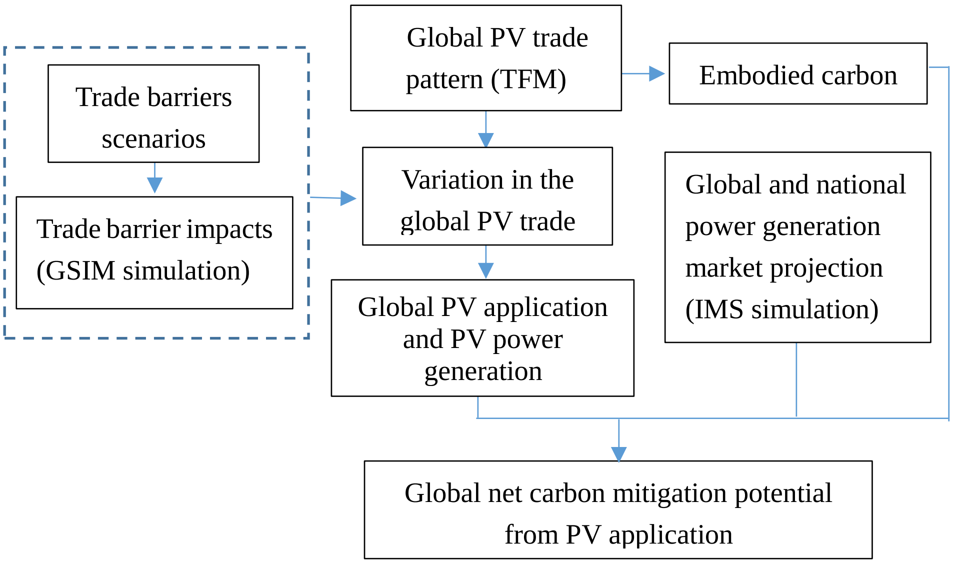

Supplementary Fig. 1 Diagram of research technical route of the research

2 Trade flows between ROW and its trade partners

2.1 Method to estimate trade flow

We estimate trade flows between ROW and its trade partners based on trade statistics. Trade value of ROW is calculated by the authors using Eq. (S1) and (S2).

$$a_{i,ROW}=a_{i,world}-\sum_j a_{i,j} \qquad \text{(S1)}$$

$$i,j \neq \text{ROW}.$$

$a_{i,ROW}$ is export value from country i to ROW, $a_{i,world}$ is export value from country i to the world.

To obtain $a_{ROW,j}$, the first step is to construct import value matrix $B_{i,j}$, whose element $b_{i,j}$, represents import trade value of i country/economy from j.

$$b_{i,ROW}=b_{i,world}-\sum_j b_{i,j} \qquad \text{(S2)}$$

$$i,j \neq \text{ROW}.$$

$b_{i,ROW}$ and $b_{i,world}$ are import values of country i from ROW and world, respectively.

In UN Comtrade database, export value of country *i* to country *j*, $a_{i,j}$ is different with import value of country *j* from country *i*, $b_{j,i}$. Normally, $a_{i,j}$ is based on FOB (Free on Board) and $b_{j,i}$ is based on CIF (Cost, insurance and Freight). Furthermore, both indicators are sourced from exporters and importers, respectively. According to trade statistics we acquired, the difference of export and import trade value of silicon, silicon wafer, as well as solar cells and modules in 2014 are all less than 5%, and those in 2017 are all less than 10%. To simplify calculation, it is reasonable to assume that the value of $a_{i,j}$ and $b_{j,i}$ are identical. It is also rational to assume that the import of each country/economy from ROW equals to the export from ROW to corresponding country/economy. In the following formula (S8), the $a_{ROW,j}$ is calculated according the share of import of country/economy *j* (IR_j) from ROW in global import from ROW.

To make the calculation more accurate, trade value flow within ROW should be deducted to compute export value from ROW to the world.

$$b_{ROW,world}=b_{world}-\sum_i b_{i,world} \tag{S3}$$

$$b_{ROW,ROW}=b_{ROW,world}\times\frac{\sum_i b_{i,ROW}}{\sum_i b_{i,world}} \tag{S4}$$

$$b_{world,ROW}=b_{world}\times\frac{\sum_i b_{i,ROW}}{\sum_i b_{i,world}} \tag{S5}$$

$$b_{world,ROW}{}'=b_{world,ROW}-b_{ROW,ROW} \tag{S6}$$

$$IR_j={b_{j,ROW}}\Big/{b_{world,ROW}{}'} \tag{S7}$$

$b_{world,ROW}$ is import value of world from ROW. b_{world} is global import value of PV trade, which is obtained from ABRAMS world trade wiki[1] and UN Comtrade[2]. $b_{world,ROW}{}'$ is import value of world from ROW with internal trade in ROW being eliminated. $b_{ROW,ROW}$ is trade value flow within ROW. IR_j is import share of country *j* from ROW in global import from ROW.

$a_{ROW,\ world}{}'$, export value of ROW to all non-ROW countries/economies, can be acquired by the same method with export trade data.

Therefore, export value from ROW to country *j*, $a_{ROW,j}$ can be obtained by Eq. (S8).

$$a_{ROW,j}=IR_j\times a_{ROW,\ world}{}' \tag{S8}$$

2.2 Re-export and re-import adjustment for PV trade flow

Difference exists in worldwide PV trade situation when entrepot trade issue is considered. In custom statistics, re-export/re-import appears in some countries/economies. For example, Hong Kong and Canada play significant roles in international cargo transit. In 2017, over 99% of solar cells and modules exported from Hong Kong were re-export, and in Canada the proportions was over 11%. Re-export and re-import would result in artificially high local trade value, displaying inaccurate trade structure. Thus, to obtain adjusted TFM, re-export trade value should be removed from total export for countries/economies acting as transshipment stations and re-allocated to its

real exporters, and re-import should also be deducted from the initial import datasets.

Based on the adjusted TFM, actual PV products export from America and East Asia exhibit small decrease compared with the initial TFM, while the actual PV products export from the rest regions, for example Europe, exhibit increase (shown in Supplementary Table 1).

Supplementary Table 1 Variation of total PV trade value after re-export/re-import problem adjustment

Region	Difference in 2017/US$ million	%/global trade value
Oceania	2.82	0.01%
Europe	180.23	0.47%
Southeast Asia	124.61	0.32%
East Asia	-147.34	-0.38%
America	-167.45	-0.43%
ROW	7.13	0.02%

Note: S Asia is the abbreviation for Southeast Asia. ROW is the abbreviation for the rest of the world.

3 Embodied carbon emission accounting

3.1 Life cycle carbon emission coefficients for PV products

We consider and refer to the status quo PV technology composition/mix of mono-crystalline silicon (35%), multi-crystalline silicon (61%) and thin-film (4%) solar cells and modules in the PV market, or refer mainly to crystalline-based PV technologies, to calculate the comprehensive carbon emission coefficients of PV products to perform carbon emissions accounting. In embodied carbon calculation, PV product life cycle carbon emission coefficients, which are obtained from Ecoinvent database, are listed in Supplementary Tables 2. When typing in a specific PV product name, e.g., silicon wafer, in the database interface, a life cycle inventory (LCI) considering emissions of all the upstream activities/processes of the product will be presented, which are used as PV product emission coefficients. However, Ecoinvent only distinguishes the data applicability for Europe and the world and claims a long applicable period (for example 1.1.2005-12.31.2020).

Life cycle inventories in the Ecoinvent are probably outdated, because recent technology improvements have resulted in lower material utilization and electricity consumption in PV product manufacturing, which also brought about reduced carbon emissions. Thus, based on LCA results from Ecoinvent database, this study considers improved technologies, including reduced silicon wafer thickness (the thickness of the mono-crystalline wafer is 270 μm and that of the multi-crystalline wafer is 240 μm according to Ecoinvent database, and the state of the art data shows that the current wafer thickness ranges from 170-180 μm [3]), reduced material usage (such as silicon, silver content in the metallization paste, copper) and kerf losses, wafer size, solar cell and module power, electricity consumption reductions, potential changes in the electricity grid mix, etc., in PV industry during the past ten years, updated the original emission coefficients of PV products [4-7],

which are listed in Supplementary Table 3. The Wind database[8] is used to obtain the international average prices of PV products in 2017. Based on the LCA data and PV products prices, the emissions coefficients of PV products are converted into emissions per unit trade value to facilitate the embodied carbon accounting based on TFM (see Data sheet 22 in Source Data).

Supplementary Table 2 Life cycle carbon emission coefficients of PV products

PV Product	Europe	The other regions	Processes/contributions included
Silicon (kg CO_2/kg silicon)	65.65	79.51	This activity ends with the production of a silicon block (a mix of monocrystalline and multicrystalline).
Silicon wafer (kg CO_2/kg wafer)	153.35	267.30	Sawing and cleaning of wafers. The process data include electricity use, water and working material consumption (e.g. stainless steel for saw-blades, argon gas, hydrofluoric and hydrochloric acid).
Solar cell (kg CO_2/W cell)	2.29	2.45	Cleaning, damage etching, texture etching, covering of backside, phosphor dotation, phosphor glass etching, printing of contacts, cleaning and quality testing.
PV module (kg CO_2/W module)	1.62	1.64	Production of the cell matrix, cutting of foils and washing of glass, production of laminate, isolation. Aluminium frame of the panel. Disposal after end of life.

Note: Life cycle carbon emissions coefficients of PV products were obtained from the Ecoinvent database. The LCA emission coefficients for each PV product are cumulative values considering all upstream processes.

Supplementary Table 3 Life cycle carbon emission coefficients of PV products with technology improvements considered

PV Product	Europe	The other regions	Processes/contributions included
Silicon (kg CO_2/kg silicon)	65.65	79.51	This activity ends with the production of a silicon block (a mix of monocrystalline and multicrystalline).
Silicon wafer (kg CO_2/kg wafer)	83.47	145.50	Sawing and cleaning of wafers. The process data include electricity use, water and working material consumption (e.g. stainless steel for saw-blades, argon gas, hydrofluoric and hydrochloric acid).
Solar cell (kg CO_2/W cell)	0.76	0.81	Cleaning, damage etching, texture etching, covering of backside, phosphor dotation, phosphor glass etching, printing of contacts, cleaning and quality testing.
PV module (kg CO_2/W module)	0.85	0.86	Production of the cell matrix, cutting of foils and washing of glass, production of laminate, isolation. Aluminium frame of the panel. Disposal after end of life.

Note: Based on LCA results from Ecoinvent database, this study considers improved technologies, including reduced silicon wafer thickness, reduced material usage (such as silicon, silver content in the metallization paste, copper) and kerf losses, wafer size, solar cell and module power, electricity consumption reductions, potential changes in the electricity grid mix, etc., to calibrate the emissions coefficients for PV products in PV industry during the past ten years, adjusts the original emission coefficients according to technology progress. The LCA emission coefficients for each PV product are cumulative values considering all upstream processes.

3.2 carbon embodied in BOS and storage system

Apart from carbon embodied in the global PV products trade, the present study also considers carbon emission related to balance of system (BOS) and storage system production. Emission coefficients of BOS and battery storage are drawn from the latest researches[9,10] (see Supplementary Table 4). BOS and storage system as corollary equipment have emission coefficients corresponding to the volumes of solar cells and modules. Thus the carbon emissions embodied in BOS and storage system can be estimated.

Finally, carbon emissions embodied in traded solar cells and modules and in the complementary BOS and storage system are all deducted from emission reduction potential of traded solar cells and modules applications to obtain net carbon reduction potential.

Supplementary Table 4 Life cycle carbon emission coefficients of BOS and storage system

Items	Carbon emission coefficient (kg CO_2-eq/kW PV system)
The Balance of system (BOS)	265
Battery storage system	204

Note: the carbon emission coefficients of BOS and storage systems was calculated by the authors based on results of up-to-date researches [8,9].

3.3 Changes in embodied carbon and the net carbon emission reduction potential of global traded PV products with respect to key parameters

Under above analysis background, this study focuses on three essential parameters, including carbon emission coefficient (EC) of PV product, PV module power conversion efficiency (CE), and performance ratio (PR) of PV system, to explore their impacts on net carbon emission reduction potential of traded solar cells and modules.

When carbon emission coefficient (EC) of each PV product decrease 10%, 20% and 30%, carbon embodied in global PV trade will drop by the equivalent percentages (see Supplementary Fig. 2 and Data sheet 23-24 in Source Data).

When decline rate of carbon emission coefficient (EC) is within 10-30%, total net carbon emission reduction of traded solar cells and modules will slightly increase by 1.22-3.66% and 0.82-2.45% in SSG and SST, respectively. Region specific net carbon emission reduction potential of traded solar cells and modules varies, and that of East Asia, which exports much more solar cells and modules than import, will increase by 7.55-22.64% and 4.69-14.06% in SSG and SST. Those of the other regions will see growth rates less than 6% in SSG and 3% in SST, respectively. (see Supplementary Fig. 3 and Data sheet 23-24 in Source Data)

When power conversion efficiency (CE) of PV system increases by 10%, 20% and 50%, solar power generation potential of traded solar cells and modules will grow by the same proportion, respectively. Along with augmented power generation potential, associated global total net carbon emission reduction will grow by 11.22-56.09% and 10.82-54.09% in SSG and SST, respectively.

Net carbon emission reduction of traded solar cells and modules in East Asia, which export much more than import, will increase to a much higher extent by 17.55-87.73% and 14.69-73.43% in SSG and SST, respectively. Those of the other regions will see growth rates up to 58.83% in SSG and 54.89% in SST, respectively. (see Supplementary Fig. 3 and Data sheet 23 in Source Data)

When performance ratio (PR) of PV system increases by 5%, 10% and 15%, solar power generation potential of traded solar cells and modules will grow by the same proportion, respectively. Along with augmented power generation potential, associated total net carbon emission reduction will grow by 5.79-17.36% and 5.53-16.58% in SSG and SST, respectively. Net carbon emission reduction of traded solar cells and modules in East Asia, will increase to a higher extent by 9.07-27.20% and 7.53-22.58% in SSG and SST, respectively. Those of the other regions will see growth rates up to 18.71% in SSG and 17.06% in SST, respectively. (see Supplementary Fig. 3 and Data sheet 23 in Source Data)

Given the above, total net carbon emission reduction potential will increase when PV products emission coefficients decrease, power conversion efficiency increases, and performance ratio rises. Conversion efficiency increase will bring about more conspicuous change in net carbon mitigation than the other two parameters.

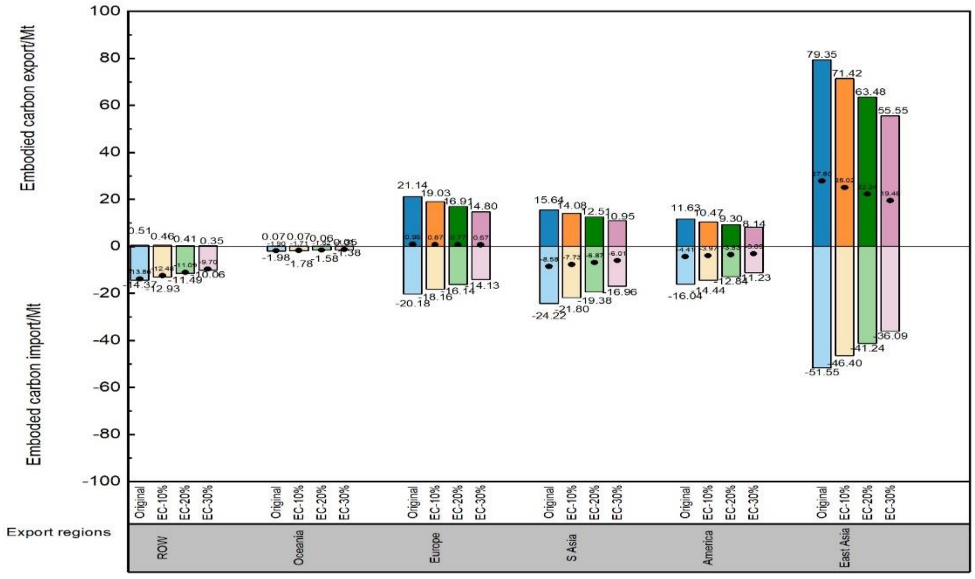

Supplementary Fig. 2 Balance of CO_2 embodied in the PV product trade when production emission coefficients (EC) decrease. These column charts show the changes in embodied carbon export, import and the balance of CO_2 embodied in the global PV product trade by regions, when production emission coefficients decrease by 10%, 20% and 30%. Bars above the horizontal axis (y=0) represent CO_2 embodied in exports, and bars under the horizontal axis represent CO_2 embodied in imports. Black dots represent the balance of embodied CO_2 ($BEET_C$). S Asia is the abbreviation for Southeast Asia. ROW is the abbreviation for the rest of the world.

a

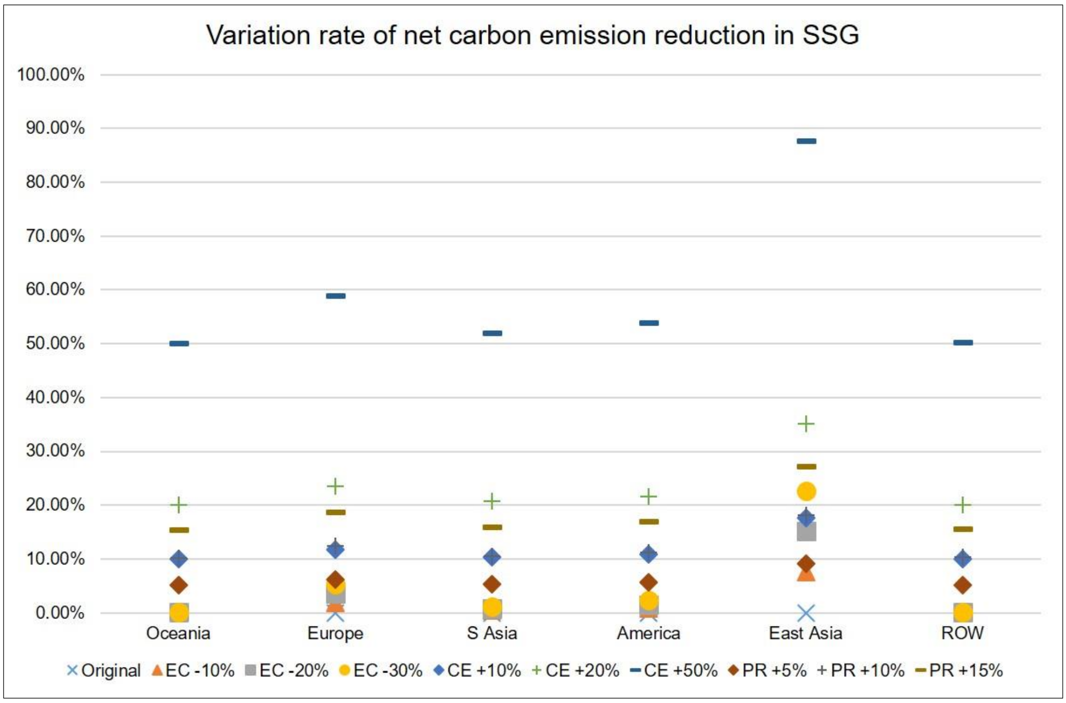

b

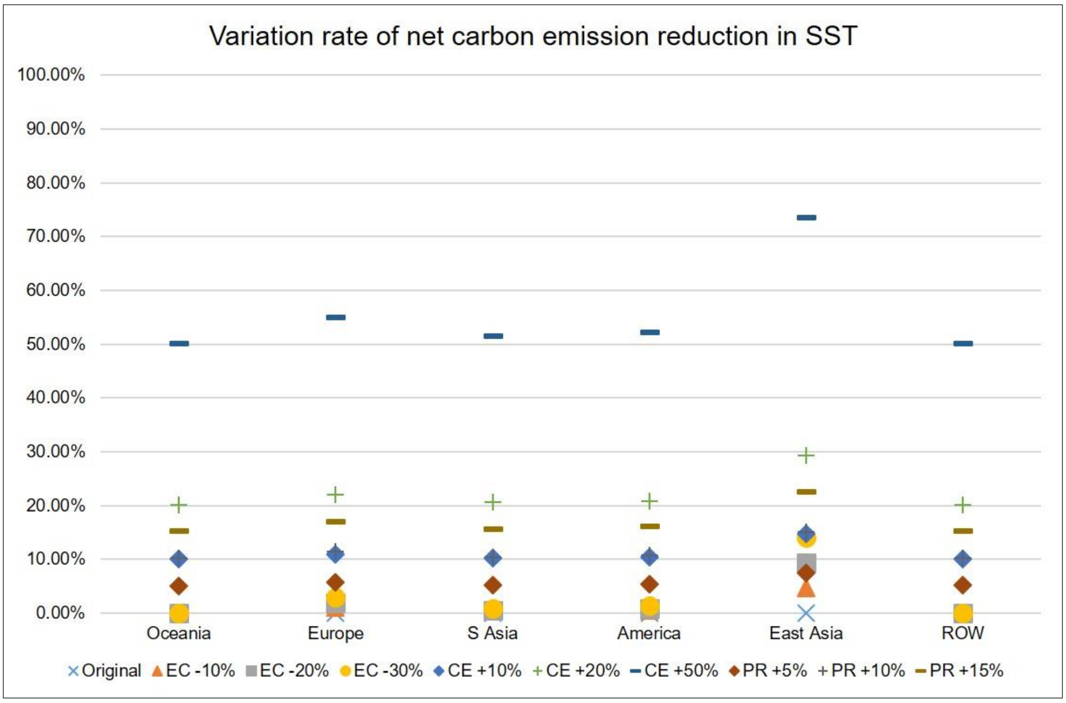

Supplementary Fig. 3 Variation rate of net carbon emission reduction potential of traded solar cells and modules when PV production emission coefficient (EC) declines, PV module power conversion efficiency (CE) increase, and performance rate (PR) increases. These figures show variation rate of net carbon emission reduction potential, when PV production carbon emission coefficient (EC) declines by 10%, 20% and 30%, conversion efficiency (CE) increase by 10%, 20% and 50%, and performance rate (PR) of PV system increases by 5%, 10% and 15% in SSG and SST, respectively. **a,b** reflects the results in SSG and SST, respectively. S Asia is the abbreviation for Southeast Asia. ROW is the abbreviation for the rest of the world.

4 Status quo solar PV power generation potential of trade-related PV application

PV application can facilitate carbon emission reduction when it is applied for local power generation replacement, especially in grids where carbon-intensive fossil fuel combustion or thermal power generation dominates. This study assumes that imported solar cells and modules are used locally to replace local power generation technologies and reduce carbon emissions. The lifetime power generation potential of traded solar cells and modules in 2017 can be calculated (see Supplementary Fig. 4).

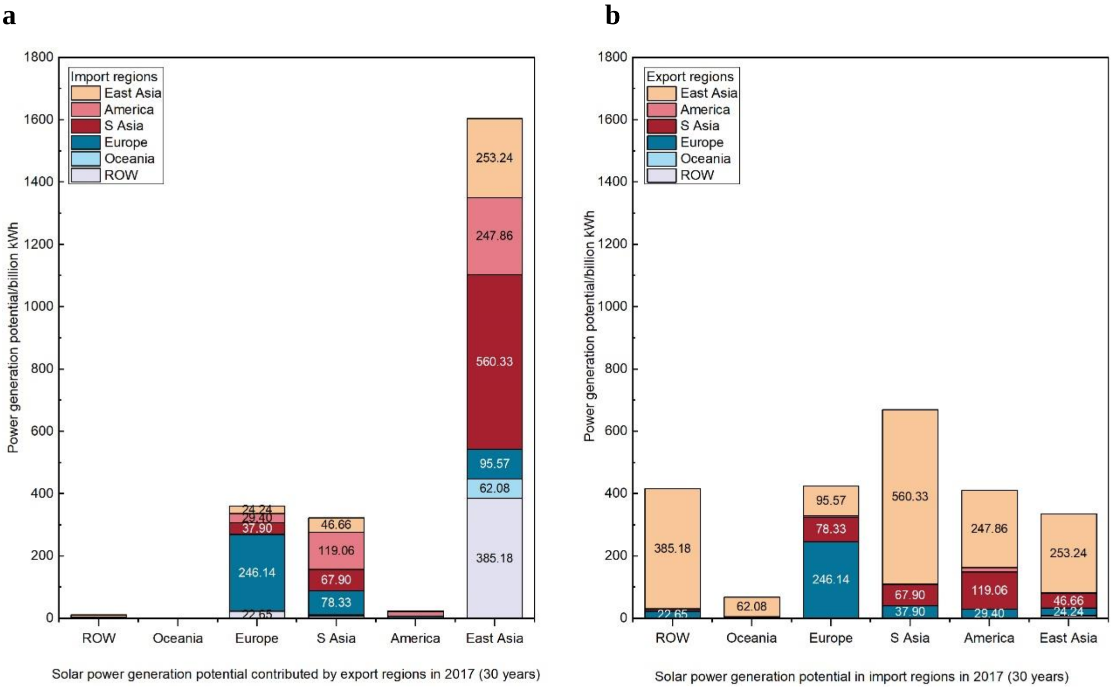

Supplementary Fig. 4 30-year lifetime power generation potential from traded solar cells and modules in 2017 The left column chart depicts contribution of each exporting region (horizontal axis) to the regional solar power generation potential, and bars represent solar power generation potentials obtained by import regions. The right column chart presents solar power generation potential received by solar cells and modules importing regions (horizontal axis), and bars exhibits contributions of export regions. S Asia is the abbreviation for Southeast Asia. ROW is the abbreviation for the rest of the world.

5 IMS model simulation

5.1 Model construction information

In this study, IMS as a powerful bottom-up dynamic energy-economy-environment (3E) model, is adopted to simulate and depict the power sector in 24 major PV application and trading partner countries/economies plus ROW-cpe from 2017-2060. Competition of solar PV with other power generation technologies are modeled based on multiple objectives and constraints, such as, total power demand, energy resource constraints, and national carbon emission reduction targets, etc., which are cited from various sources, including national energy development outlooks, NDCs target commitments, long-run energy strategic plans and decarbonisation pathway research reports of

various countries/economies (see Supplementary Table 5).

Supplementary Table 5 Country/economy-specific long-run power market and supply information for IMS model construction

Country or Economy	Sources	Key information
Australia	ClimateWorks Australia[11]	'1.5C All-in' scenario: this scenario models an emissions outcome compatible with a limiting global temperature rise to 1.5 degrees Celsius. This scenario stays within the 50% probability of the 1.5 degrees Celsius carbon budget for Australia (achieving net zero by 2035), and then overcompensates with net-negative emissions through to 2050 to improve the chances of achieving this goal.
Austria	Bundesministerium Klimaschutz, Umwelt, Energie, Mobilität, Innovation und Technologie[12]	According to projection, in 2040, the total electricity generation is 81TWh, among which solar PV accounts for 6.17%, hydro power accounts for 54.32%, onshore wind accounts for 19.75%, and biofuels accounts for 7.4%.
Belgium	The Belgian Federal Climate Change Section of the Federal Public Service Health, Food Chain Safety and Environment[13]	'EU integration' scenario: reaches 87% GHG reduction. This scenario assumes that European electricity grids are strongly developed, European energy markets are highly integrated with share infrastructure, and energy system is largely based on renewable primary energy sources.
Brazil	SDSN-IDDRI[14]	Under pathways to the deep decarbonization in Brazil, the 2050 GHG emissions is 70% lower than that in 2010. As to energy sector in 2050, share of solar power in power generation grows to 11.3%, biomass-based thermopower is nearly 20%, hydropower is little over 50%. The complete replacement of natural gas by biofuels, the last fossil fuel still in use for power generation by 2030, is completed.
Canada	Canada Energy Regulator[15]	Evolving Energy System scenario: this scenario considers the impact of continuing the historical trend of increasing global action on climate change throughout the projection period, assumes a stronger shift towards wind, solar, and other low carbon technologies and lower demand for fossil fuels.
China	Institute of Energy Environment and Economy, Tsinghua University[16]	2060 Carbon-neutral scenario: carbon emission will peak before 2030, and seek to achieve carbon-neutral before 2060. In 2060, power generation is almost all from renewable energy, especially wind and solar power, occupying about 50% of total amount.

Czechia	McKinsey & Company[17]	According to cost-optimal pathway, Czech will reach net-zero by 2050, large capacities of solar, wind, and CCS-equipped gas will be added by 2050, whose shore in total power generation will approximate 24.43%, 14.36% and 16.60%.
France	SDSN-IDDRI[18]	Diversification (DIV) pathway: share of nuclear power in electricity mix stabilizes at 50% after 2025, 40% share of renewables in the electricity mix in 2050. To achieve 75% reduction in GHG emissions in 2050, This scenario yields a 24% reduction in demand, a 67% energy-decarbonization need to be achieved
Germany	Umweltbundesamt[19]	GreenE2 scenario: all GHG emissions will at least reduce by 95% by 2050. In energy mix, shares of PV, wind, hydropower are nearly 22.39%, 70.93% and 3.42%.
India	SDSN-IDDRI[20]	Sustainable deep decarbonization scenario: this scenario assumes a strong push for India's large renewable energy potential and using CCS along with coal. A deeper decarbonization is supposed, which is 33% lower than in the conventional scenario in 2050 (about 2108 Mt).
Italy	SDSN-IDDRI[21]	The Demand Reduction scenario (DMD_RED): 80% GHG emission reduction by 2050 compared to 1990, models the response of the energy system to a limited availability/commercialization of CCS(especially in the industrial sector) and a high cost of decarbonization, variable renewable energies account for over 50% of total power generation in 2050.
Japan	SDSN-IDDRI[22]	Limited Carbon Capture and Sequestration (CCS) scenario: achieve 80% reduction of GHG by 2050 compared to 1990 under continued economic growth, three strong actions include large-scale energy demand reduction by energy-efficiency measures, extensive diffusion of low-carbon electricity in end-uses, and certain carbon sequestration level.
Malaysia	Academy of Sciences Malaysia[23]	Scenario 1-Carbon free energy mix in electricity generation: fossil fuels in power generation will be completely phased out by 2050, and replaced by eight types of alternative resources in energy mix, the proposed energy mix will achieve net-zero in electricity sector between 2035 and 2040. In 2050, total carbon avoidance is about 217 Gt.
Mexico	SDSN-IDDRI[24]	No-CCS alternative scenario: reducing GHG emissions to 50% below 2000 levels by 2050 in a pathway that does not include CCS, zero-carbon power generation must be in place by 2025, especially solar photovoltaic and geothermal sources will reach their maximum potential.

Netherlands	PBL Netherlands Environmental Assessment Agency[25]	'Binnenlands duurzaam' scenario: focuses on sustainable park and large scale electrification, GHG emission will be reduced by 95% by 2050, renewable power will grow apparently. In energy mix, PV and offshore wind will make up over 35% and 20% of total power generation.
Poland	McKinsey & Company[26]	Carbon neutrality will be achieved by 2050, cutting GHG emissions by 91% from 2017 to 2050, increasing carbon sinks to abate the rest 9% emissions, employing energy efficiency and switching from carbon-based fuels to zero-emissions energy sources.
Korea	Climate Action Tracker[27]	According to the 8th electricity plan, the Korean electricity mix by 2030 will include coal (36.10%), natural gas (18.80%), nuclear (23.90%), and renewable energy (20%).
Russia	International Network for Sustainable Energy[28]	To achieve a sustainable energy vision, renewable energy used as primary energy in Russia will increase to 50% in 2040 and to over 90% in 2050, share of renewable energy power is above that in primary energy. Nuclear energy will be phased out until 2030, fossil fuel use will be phased out until 2050. In 2050, carbon emission reduction from energy consumption will be 90% lower than that in 2000.
Taiwan Province	Tsai M-S, Chang S-L[29]	Case 2000_50 scenario: carbon emissions reduced by 50% relative to 2000 level, accumulated installed capacity of renewable energy will reach 8,921 MW in 2025-2050, coal-fired power, steel and iron industry, cement industry will be installed with CCS from 2025.
Thailand	WWF[30]	Advanced Sustainable Energy Scenario (ASES): 70% renewable generation by 2030, 90% by 2040, 100% by 2050, realize zero emissions in 2050, uptake of electric vehicles is assumed to double by 2050, By 2050, solar PV will have the highest share of 31%, then biomass 28%.
Turkey	Kilickaplan A, Bogdanov D, Peker O, et al.[31]	Power sector scenario: to realize 100% renewable energy system and coal power will be completely phased out by 2050 in Turkey. Three main technologies are involved: technologies for renewable electricity, energy storage technologies and energy sector bridging technologies.
United Kingdom	Department for Business Energy & Industrial Strategy, Government of UK[32]	Reference scenario: to achieve emissions reduction to net zero by 2050, low carbon share of UK electricity generation will rise from 51% in 2018 to 83% in 2040, natural gas generation will fall rapidly until the late 2020s. Government policies which have been implemented, adopted, or planned as at August 2019 are considered.
USA	U.S. Energy Information	AEO2020 Reference case: represents EIA's best assessment of U.S. energy market through 2050. Total renewable generation

	Administration[33]	exceeds natural gas-fired generation after 2045, natural gas-fired generation is the marginal fuel to fulfill incremental demand and increases by 0.8% per year through 2050, coal-fired and nuclear generation decline through the mid-2020s, and stabilize over the longer term.
Vietnam	the Electricity and Renewable Energy Authority in Vietnam, the Danish Energy Agency[34]	C4 Combination scenario：the most ambitious scenario to achieve GHGs emission reduction under the Paris Agreement and green energy transition. Share of renewable energy power will be 43% by 2050, GHG emission reduction will reach 45% compared to BAU (a scenario from Ministry of Natural Resources and Environment) by 2050, the coal restriction from 2025 and the high uptake of energy efficient technologies are expected.
World	IRENA[35]	Remap case: to keep average global temperatures well below 2°C and ideally limit warming to 1.5°C compared to pre-industry levels, annual energy-related carbon emissions decline 70% below 2019's level, which need to peak in 2020 and decline thereafter, reduce by about 3.5% per year from now until 2050. Renewable energy sharing in power generation need to be 75% by 2040, and 86% by 2050, accelerated deployment of solar and wind will be seen. Total fossil fuel reduction rate will be 64% by 2050 compared to current level.

5.2 Projection to electricity power production composition in 25 countries/economies

According to IMS simulation, each country/economy will experience considerable energy transition from 2017-2060, whose power sector will mostly shift from fossil fuel combustion electricity dominated structure to remarkable renewable energy power generations, especially solar and wind power (see Supplementary Fig. 5).

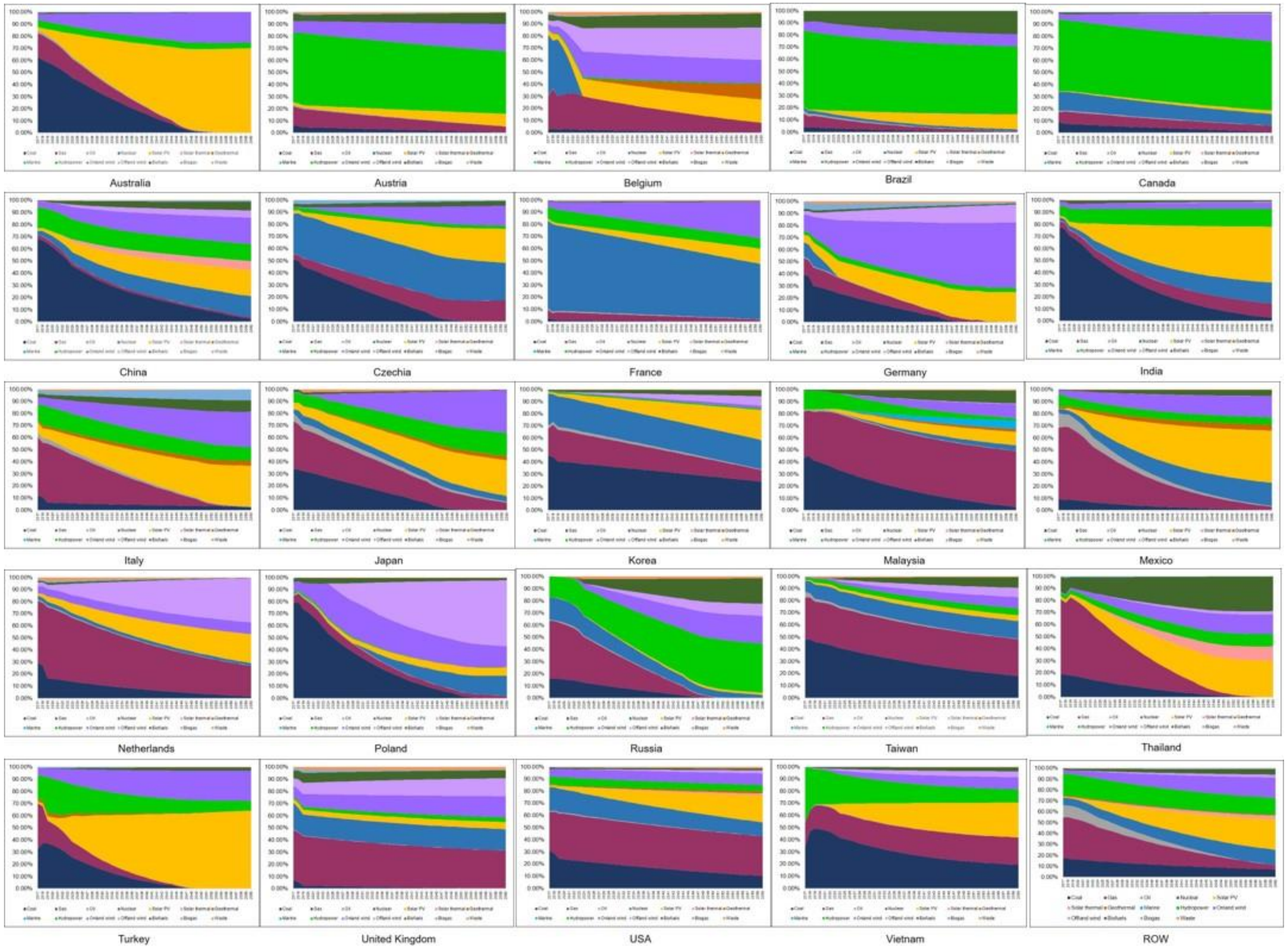

Supplementary Fig. 5 Projection to electricity power production composition in 25 countries/economies from 2017 to 2060. ROW is the abbreviation for the rest of the world.

5.3 Comparison of global and country/economy power market projection using IMS and TIMES model

The TIMES (The Integrated MARKAL and EFOM System) is a combination of the MARKAL (Market Allocation Model) and EFOM (Energy Flow Optimization Model), which is developed and maintained by the ETSAP (Energy Technological System Analysis Program) of IEA (International Energy Agency) [36]. It is a technology rich bottom-up tool to assess the optimal energy flow and mix of technologies, with the objective function of cost minimization [37].

The present study adopted TIMES model to simulate the energy structure of power market/supply considering the current policies of each involved country/economy. The results are compared with IMS simulation results to test the robustness and reliability of IMS simulation results. Identical to IMS simulation, we model the top 24 countries/ economies in terms of PV products trade volume as well as the rest of world (ROW_{-CPE}), with a time horizon from 2015-2060 and a five-year reporting period. For the TIMES modelling, we apply the same economic parameters, environment parameters and technology characteristics as in IMS. The power generation technologies in TIMES model also include coal power, natural gas power, oil power, onshore and offshore wind power, marine energy, solar PV and concentrating solar power, hydro power, bioenergy, geothermal power and so on. Models for all countries/economies are calibrated using various data sets, such as IEA, IRENA, BP and other official announcements. The simulation results of TIMES model are presented in Supplementary Fig. 6. The simulation results of TIMES model and IMS model are compared and presented in Data sheet 25 in Source Data. In general, the difference between the two model simulations are within 10%, indicating a high reliability of the IMS model simulation results.

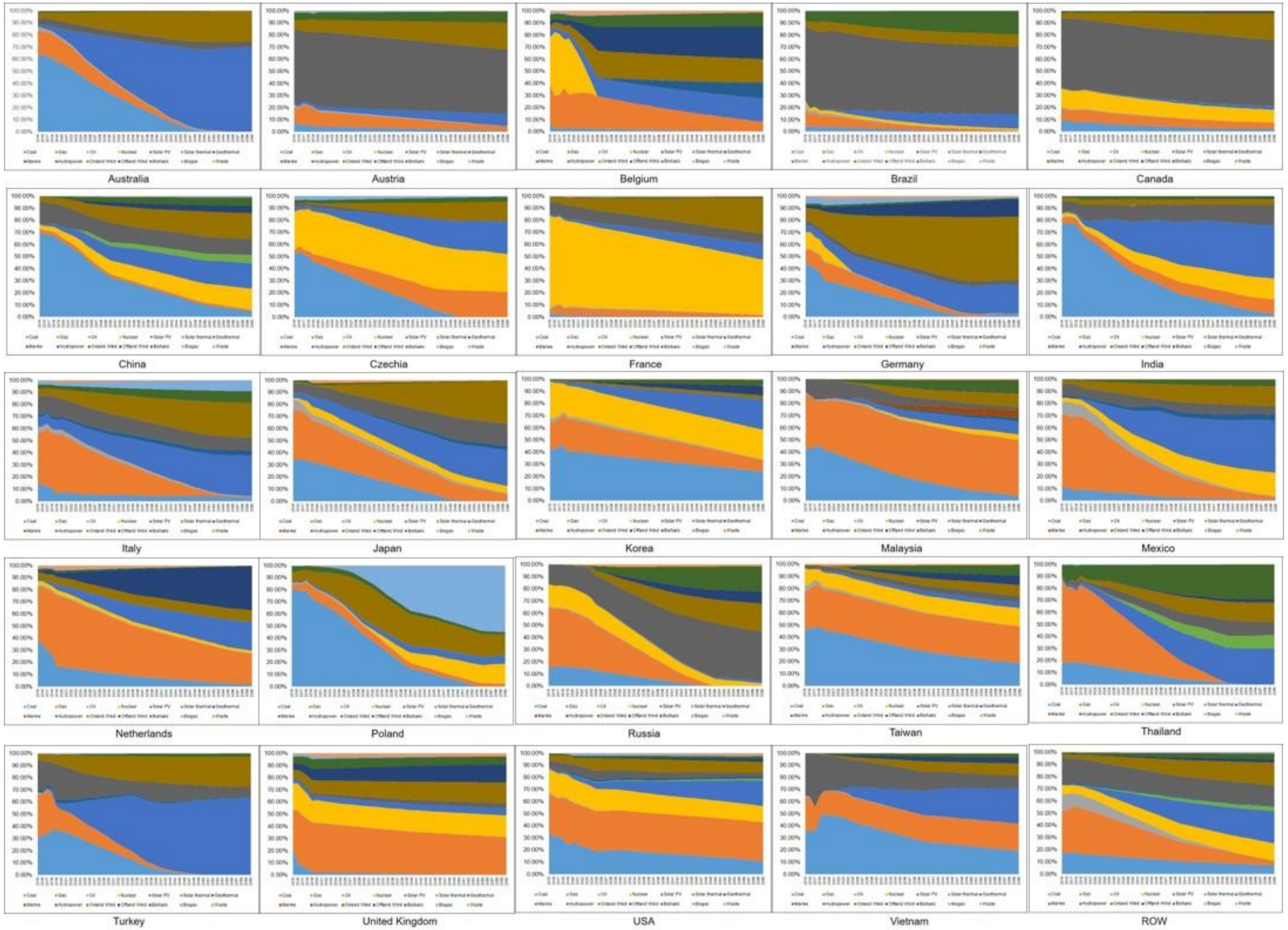

Supplementary Fig. 6 TIMES model projection to electricity power production composition in 25 countries/economies from 2017 to 2060. ROW is the abbreviation for the rest of the world.

6 GSIM model simulation

6.1 GSIM model construction information

Trade barriers consist of ordinary tariffs and non-tariff measures (NTMs). Apart from ordinary tariffs, antidumping measures and countervailing measures are quite frequently used in global PV products trade frictions or conflicts, which belong to NTMs. In GSIM modelling, three trade barrier scenarios are simulated and compared with business-as-usual (BAU) scenario to test the impacts of barriers on solar cells and modules trade (see Supplementary Table 6). 24 countries/economies ranked top PV product trader partners in 2017 are selected, the other countries/economies are merged as ROW-$_{CPE}$ (see Supplementary Table 7). Hong Kong is incorporated in China in GSIM model simulation. There have been recent efforts to estimate the substitution elasticity as the HS6 level, e.g., Soderbery[38] has a mean substitution elasticity of over 3, Fontagné et al.[39] have a substitution elasticity centered around 5. Elasticities of solar cells and modules trade for the current study are referred from the latest study, which adopts a double logarithmic model to estimate Armington substitution elasticity of solar cell and module trade tailored for GSIM model. [40] (see Supplementary Table 8). Tariff rates are obtained from the WTO[41] and MOFCOM[42] (Data sheet 14 in Source Data). Ad-valorem equivalents (AVEs) of NTMs relative to solar cells and modules in 2015, which are corresponding to HS code 854140, are obtained from the latest research[43] and applied in GSIM model scenario settings.

Supplementary Table 6 Scenario settings in trade barrier impacts analysis

Scenario name	Detailed information
BAU	The BAU scenario is set according to status quo ordinary tariffs, antidumping and countervailing measures imposed on global traded solar cells and modules in 2017. In the BAU scenario, AVEs of NTMs in 2015 from each economy are drawn from the latest research[43]. To match actual PV trade barriers in 2017, the other two issues are included: Turkey has imposed antidumping tariffs on imported Chinese solar cells and modules since 2017; Brazil has imposed Most-Favored-Nation (MFN) duties on imported solar cells and modules from all other countries/economies.
TBS0	The reduced trade barrier scenario (TBS0) assumes that 50% of both tariff and NTMs are removed, which is designed to test whether freer trade environment can facilitate global PV trade, application and carbon reduction.
TBS1	The foreseeable higher trade barrier scenario (TBS1) on top of BAU, where the USA-China trade war and trade protection measures of India in 2018 are considered. The USA imposes safeguard measures under section 201 to solar cells and modules from all origins (the special tariff rate is set to 30% as the first year standard)[44], and imposes safeguard measures under section 201 and 301 on China mainland products[44-47]. China imposes a 25% retaliation tariffs on the USA products[42]. India imposes safeguard measures on imported solar cells and modules from China and Malaysia with a tariff rate of 25%[42].
TBS2	The intensified trade barrier scenario (TBS2) is based on recent business reports that, a worsened multilateral trade protection proposition can involve that, European countries reinstate antidumping and countervailing measures on Chinese PV product and expand to solar cells and modules from all origins; Brazil keeps tariff on solar cells and modules from all origins; India imposes safeguard measures on imported solar cells and modules from all origins; Turkey keeps imposing antidumping tariffs on Chinese solar cells and modules; and the USA imposes 201 tariff on Chinese solar cells and modules and expand to those from all origins, and China imposes retaliation tariffs on the USA solar cells and modules[48].

Note: For TBS, the antidumping and countervailing tariff rate of the EU is set to 47.7% based on the European Commission determination[49,50]. The safeguard duty of the USA is set to 30% based on Section 201[44], while a higher rate is imposed on Chinese PV products considering the superposition of Section 201, Section 301, and antidumping and countervailing policies[44,46,47]. The other tariff and NTMs rates are set based on the WTO[41], MOFCOM[42], official statistics[48,51] or business news reports[52].

Supplementary Table 7 Major trade partner countries/economies in GSIM model

Country/Economy	Abbreviation	Country/Economy	Abbreviation
Australia	AUS	Mexico	MEX
Austria	AUT	Netherlands	NLD
Belgium	BEL	Taiwan	TWN
Brazil	BRA	Poland	POL
Canada	CAN	Korea	KOR
China	CHN	Russia	RUS
Czechia	CZE	Thailand	THA
France	FRA	Turkey	TUR
Germany	DEU	United Kingdom	GBR
India	IND	USA	USA
Italy	ITA	Vietnam	VIE
Japan	JPN	Countries/Economies other than the 24 largest PV product trade partners	ROW_{-CPE}
Malaysia	MYS		

Note: countries/economies other than the 24 largest PV product trade partners are merged as ROW_{-CPE}.

Supplementary Table 8 Setting s of elasticities of solar cells and modules trade in GSIM model

Country/economy	AUS	AUT	BEL	BRA	CAN	CHN	CZE	FRA	DEU
Demand Elasticity	-0.75	-0.75	-0.75	-0.75	-0.77	-0.94	-0.75	-0.83	-0.83
Supply Elasticity	2.36	2.36	2.36	2.36	2.94	4.01	2.36	3.38	3.38
Substitution Elasticity	2.76	2.76	2.76	2.76	2.76	2.76	2.76	2.76	2.76
Country/economy	ITA	JPN	MYS	MEX	NLD	TWN	POL	KOR	IND
Demand Elasticity	-0.75	-0.81	-0.71	-0.68	-0.82	-0.64	-0.75	-0.74	-0.75
Supply Elasticity	2.36	2.47	2.95	2.87	2.75	3.23	2.36	2.95	2.36
Substitution Elasticity	2.76	2.76	2.76	2.76	2.76	2.76	2.76	2.76	2.76
Country/economy	RUS	THA	TUR	GBR	USA	VIE	ROW_{-CPE}		
Demand Elasticity	-0.75	-0.75	-0.75	-0.87	-0.77	-0.71	-0.75		
Supply Elasticity	2.36	2.36	2.36	2.47	2.94	2.95	2.36		
Substitution Elasticity	2.76	2.76	2.76	2.76	2.76	2.76	2.76		

Note: Elasticities of solar cells and modules trade are referred from the latest study[40]. Countries/economies other than the 24 largest PV product trade partners are merged as ROW_{-CPE}.

6.2 Impacts of trade barrier on PV products price

If half of trade barriers on traded solar cells and modules are removed (TBS0), countries/economies who are exerting trade barriers on solar cells and modules imports will all see consumer prices decline ranging from 0.41-22.50%, owing to growing foreign supply.

If trade barriers on PV products are intensified (TBS1 and TBS2), countries/economies who exert high tariffs and NTMs will witness increased consumer price of solar cells and modules because of reduced import. Based on BAU, when the USA-China trade war and trade protection measures of India in 2018 are considered (TBS1), the USA and India will see consumer prices of solar cells and modules surge by 15.46% and 11.13%, respectively, while those in all the rest countries/economies will decrease slightly by 0.05-0.74%. When the intensified trade barrier scenario (TBS2) are taken into consideration, countries/economies imposing higher trade barriers will suffer 11.61-22.36 % higher consumer price compared with BAU, even worse than in TBS1, and the rest countries/economies will enjoy slight consumer price reduction, ranging from 0.56-1.27 % (see Supplementary Fig. 7).

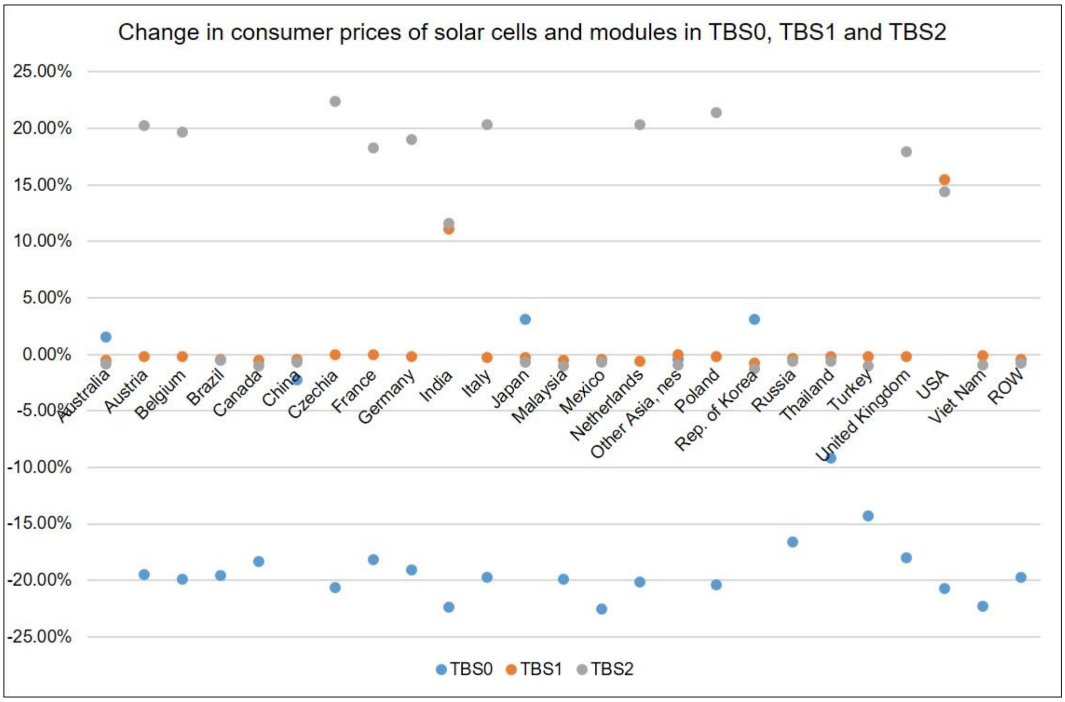

Supplementary Fig. 7 Change in consumer prices of solar cells and modules in TBS0, TBS1 and TBS2. This figure reflects consumer prices variation rates of solar cells and modules in 25 economies under TBS0, TBS1 and TBS2. ROW is the abbreviation for the rest of the world.

6.3 Sensitivity of GSIM modelling results to substitution elasticity setting

In the GSIM model simulation, a sensitivity analysis is conducted to test the changes in solar cells and modules imports and consumptions with substitution elasticity (SE) value ranging from -20% to +20% under trade barrier reduction scenario and the two intensified trade barrier scenarios.

This variation rate index Sen_{SE} is constructed shown by Eq. (S9) and (S10).

$$Q_{i,TBS}=Q_{i,BAU}+\Delta Q_{i,TBS} \tag{S9}$$

$$Sen_{SE}=\left.[\left(Q_{i,TBS\text{-}SE}\right)\text{-}(Q_{i,TBS})]\right/Q_{i,TBS}=\left.\Delta Q_{i,TBS\text{-}SE}\right/Q_{i,TBS} \tag{S10}$$

$Q_{i,TBS}$ is total solar cells and modules import or consumption quantity in country/economy i under TBS scenarios when SE is set to original value. $Q_{i,BAU}$ is total solar cells and modules import or consumption quantity in country/economy i under BAU. $\Delta Q_{i,TBS}$ represents variation quantity of import or consumption in country/economy i under TBS scenarios. $Q_{i,TBS\text{-}SE}$ is total solar cells and modules import or consumption quantity in country/economy i under TBS scenarios with changed SE. $\Delta Q_{i,TBS\text{-}SE}$ represents variation quantity of import or consumption in country/economy i under TBS scenarios when SE changes.

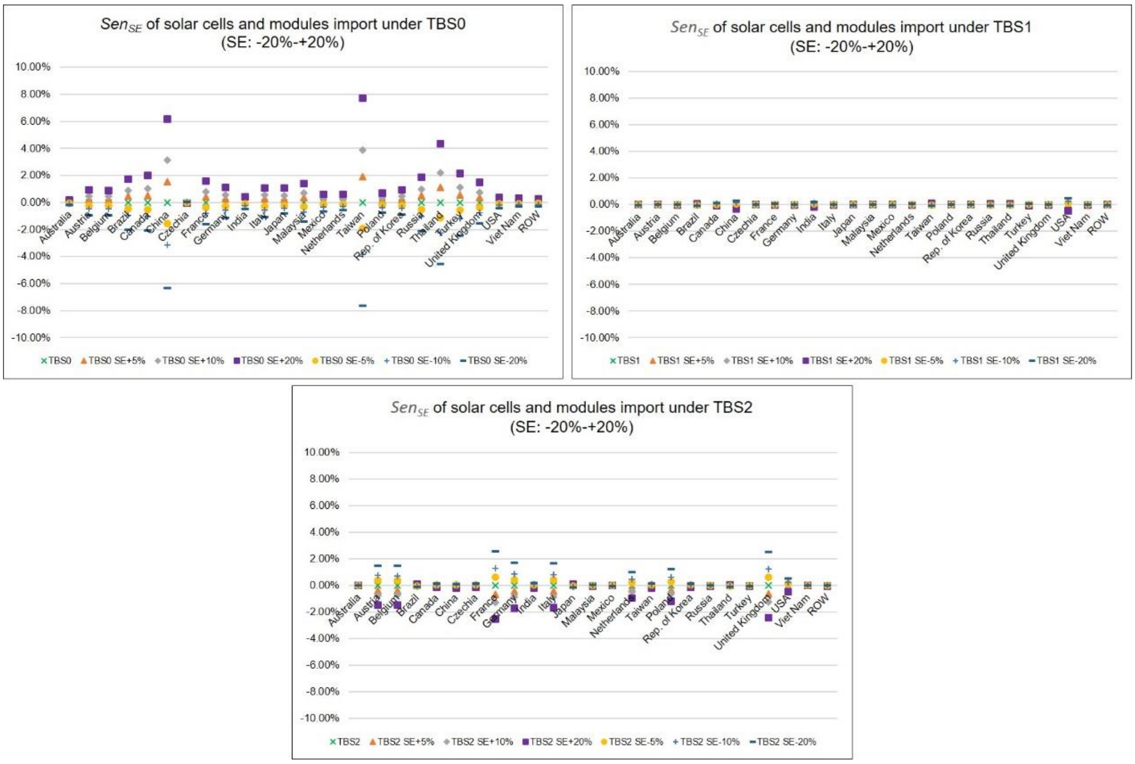

Supplementary Fig. 8 Sensitivity (Sen_{SE}) of solar cells and modules import in 25 countries/economies under TBS0, TBS1 and TBS2 with substitute elasticity (SE) ranging from -20% to +20%. ROW is the abbreviation for the rest of the world.

When SE varies by -20% to +20% under TBS0, TBS1 and TBS2, import and consumption variation directions/trends of most countries/economies will keep the same as under the original SE values. The changing rate (Sen_{SE}) of import in most countries/economies will be within 4.0% under TBS0, will be within 2.0% under TBS2, and will be almost neglectable under TBS1, indicating that the sensitivity of import with regard to SE change is small. Under TBS0, most countries/economies

will have higher imports augment when SE increases, and lower import when SE decreases. Under TBS1 and TBS2, when SE increases, most countries/economies will reduce imports by less than 0.5% and 1.0%, respectively. The situation will be the opposite when SE declines (see Supplementary Fig. 8 and Data sheet 26 in Source Data).

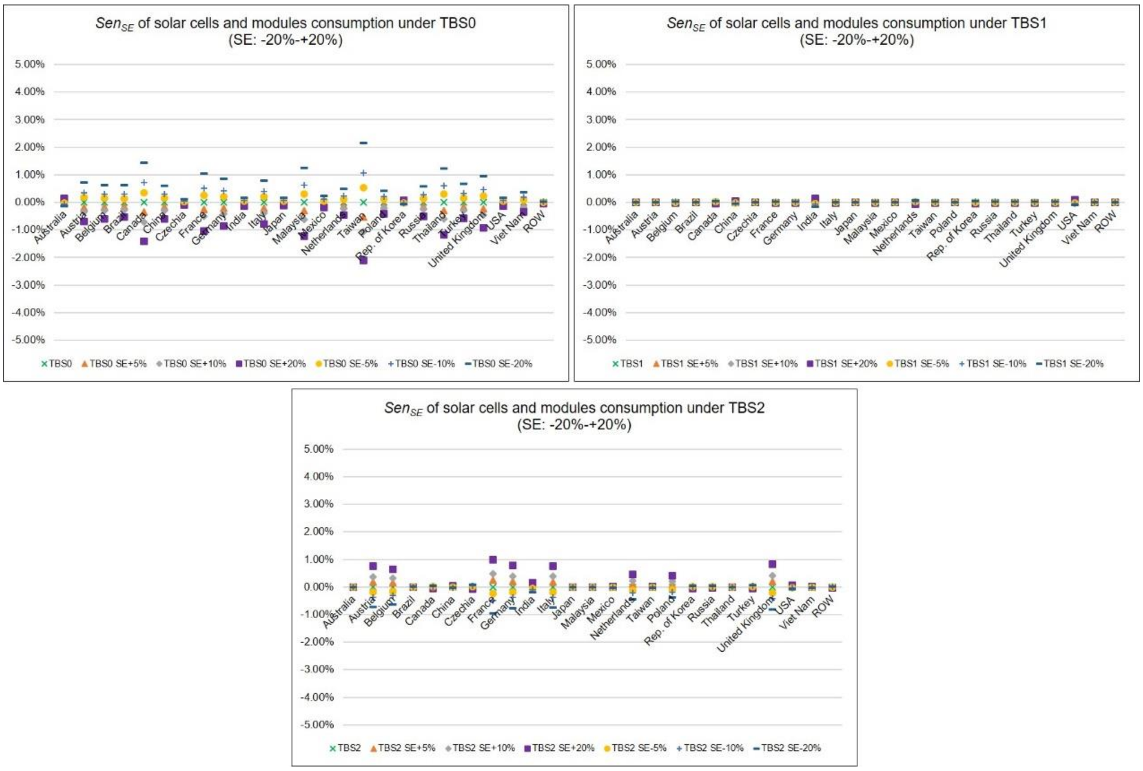

Supplementary Fig. 9 Sensitivity (*Sen_{SE}*) of solar cells and modules consumption in 25 countries/economies under TBS0, TBS1 and TBS2 with substitute elasticity (SE) ranging from -20% to +20%. ROW is the abbreviation for the rest of the world.

The Sen_{SE} of consumption in most countries/economies will be within 2.0% under TBS0, will be within 1.0% under TBS2, and will be almost neglectable under TBS1. This also indicates that the sensitivity of consumption with regard to SE change is even smaller than that of import. (see Supplementary Fig. 9 and Data sheet 26 in Source Data).

The lack of sensitivity of solar cells and modules trade and consumptions to SE provides support of robustness and reliability of the GSIM model simulation results, and the subsequent estimation of the policy shock impacts on the trade related solar PV power generation potential and carbon emission reduction of PV application.

7 Additional discussion

Trade pattern is an important factor relevant to the projection results. We consider that trade pattern is largely decided by the differences in factor endowment of various countries/economies

which are relatively stable, e.g., though East Asia countries including China have the problem of aging population and rapidly rising middle-class, their comparative advantages in PV goods supply over other regions should last long, and thus we assume a stable trade pattern to facilitate the trade barrier scenario analysis. For the power demand predictions for various countries/economies in IMS model simulations, the population and income factors have been considered in the parameter setting.

In this study, we would like to disclose the fact/rule that, ongoing, foreseeable and possible trade barriers can impact global PV trade and harm the global carbon mitigation capacity, and try to call on for freer trade of PV goods. We and many scholars are very much concern that trade war is not simply a temporary phenomenon, but a function of the current and long-lasting geopolitical environment in which the USA-Sino competitions are intensified, which is not conducive to global joint effort fighting against climate change. Recently, major carbon emission countries/economies, including China, the USA, Japan, EU, among others, made serious promise to achieve carbon neutrality by 2050 or 2060. We would like to take these promises as serious ones and have taken these factors into IMS simulation, referring to national energy plans, energy development outlooks, NDC target commitments, long-term energy strategic plans and decarbonisation pathway research reports of various countries/economies.

References

1 ABRAMS. *ABRAMS world trade wiki*, <https://en.abrams.wiki/tools/marketintelligence?utm_source=partner&utm_medium=website&utm_campaign=uncomtrade> (2019).

2 UN. *UN Comtrade Database*, <https://comtrade.un.org> (2018).

3 Frischknecht, R., Stolz, P., Krebs, L., Wild-Scholten, M. d. & Sinha, P. Life cycle inventories and life cycle assessments of photovoltaic systems 2020. (IEA-PVPS, 2020).

4 IEA-PVPS. Trends in photovoltaic applications 2018 (2018).

5 IEA-PVPS. Trends in Photovoltaic applications 2019. (2019).

6 IEA-PVPS. Trends 2017 in photovoltaic applications. (2017).

7 CPIA. China Photovoltaic Annual Report 2017-2018. (2018).

8 Wind. (2019).

9 Leccisi, E., Raugei, M. & Fthenakis, V. The Energy and Environmental Performance of Ground-Mounted Photovoltaic Systems—A Timely Update. *Energies* **9**, doi:10.3390/en9080622 (2016).

10 Raugei, M., Leccisi, E. & Fthenakis, V. M. What Are the Energy and Environmental Impacts of Adding Battery Storage to Photovoltaics? A Generalized Life Cycle Assessment. *Energy Technology* **8**, 1901146, doi:https://doi.org/10.1002/ente.201901146 (2020).

11 Butler, C. *et al.* Decarbonisation futures: Solutions, actions and benchmarks for a net zero emissions Australia. (ClimateWorks Australia, 2020).

12 Bundesministerium Klimaschutz umwelt energie mobilität innovation und technologie.

Integrierter nationaler energie-und climaplan für Österreichs. (Bundesministerium Klimaschutz, umwelt, energie, mobilität, innovation und technologie, 2019).
13 Michel, C. *et al.* Scenarios for a low carbon Beligum by 2050. (The Belgian Federal Climate Change Section of the Federal Public Service Health, Food Chain Safety and Environment, 2013).
14 Rovere, E. L. L., Gesteira, C., Grottera, C. & Wills, W. Pathways to deep decarbonization in Brazil. (SDSN-IDDRI, 2015).
15 Canada Energy Regulator. Canada's energy future 2020: Energy supply and demand projections to 2050. (Canada Energy Regulator,, 2020).
16 Zhang, X. Decarbonisation and energy transition pathway analysis under carbon neutral goal in 2060. (Institute of Energy Environment and Economy, Tsinghua University, 2020).
17 Viktor, H., Vít, J., Bram, S. & Daniel, S. Pathways to decarbonize the Czech Republic: carbon-neutral Czech Republic 2050. (McKinsey & Company, 2020).
18 Criqui, P. & Hourcade, J.-C. Pathways to deep decarbonization in France. (SDSN-IDDRI 2015).
19 Monika, D. *et al.* Transformationsprozess zum treibhausgasneutralen und ressourcenschonenden Deutschland – Vergleich der Szenarien. (Umweltbundesamt, 2020).
20 Shukla, P. R., Dhar, S., Pathak, M., Mahadevia, D. & Garg, A. Pathways to deep decarbonization in India. (SDSN-IDDRI, 2015).
21 Virdis, M. R. *et al.* Pathways to deep decarbonization in Italy. (SDSN-IDDRI, 2015).
22 Kainuma, M., Masui, T., Oshiro, K. & Hibino, G. Pathways to deep decarbonization in Japan. (SDSN-IDDRI, 2015).
23 Academy of Sciences Malaysia. Carbon free energy: roadmap for Malaysia. (2015).
24 Tovilla, J. & Buira, D. Pathways to deep decarbonization in Mexico. (SDSN-IDDRI, 2015).
25 De Rijksoverheid. Voor Nederland. Richting 2050: systeemkeuzes en afhankelijkheden in de energietransitie. (2018).
26 Engel, H., Purta, M., Speelman, E., Szarek, G. & Pluijm, P. v. d. Carbon-neutral Poland 2050: Turning a challenge into an opportunity. (McKinsey & Company, 2020).
27 Climate Action Tracker. *South Korea: current policy projections*, <https://climateactiontracker.org/countries/south-korea/current-policy-projections/> (2020).
28 INFORSE-Europe. Vision for sustainable energy for Russia. (2008).
29 Tsai, M.-S. & Chang, S.-L. Taiwan's 2050 low carbon development roadmap: An evaluation with the MARKAL model. *Renewable and Sustainable Energy Reviews* **49**, 178-191, doi:https://doi.org/10.1016/j.rser.2015.04.153 (2015).
30 WWF. Thailand power sector vision 2050: toward 100% renewable energy by 2050. (2016).
31 Kilickaplan, A. *et al.* An energy transition pathway for Turkey to achieve 100% renewable energy powered electricity, desalination and non-energetic industrial gas demand sectors by 2050. *Solar Energy* **158**, 218-235, doi:https://doi.org/10.1016/j.solener.2017.09.030 (2017).
32 Department for Business Energy & Industrial Strategy. Government of UK. Updated energy and emissions projections 2019. (2020).
33 U.S. Energy Information Administration. Annual Energy Outlook 2020 with projections to 2050. (2020).
34 EREA & DEA. Vietnam energy outlook report 2019. (2019).
35 IRENA. Global Energy Transformation: A Roadmap to 2050. (2018).
36 Richard, L. & Maryse, L. ETSAP-TIAM: the TIMES integrated assessment model Part I: Model structure. *Computational Management Science* **5**, 7-40, doi:10.1007/s10287-007-0046-z

(2008).
37 Huang, W., Chen, W. & Anandarajah, G. The role of technology diffusion in a decarbonizing world to limit global warming to well below 2 °C: An assessment with application of Global TIMES model. *Applied Energy* **208**, 291-301, doi:https://doi.org/10.1016/j.apenergy.2017.10.040 (2017).
38 Soderbery, A. Trade elasticities, heterogeneity, and optimal tariffs. *Journal of International Economics* **114**, 44-62, doi:https://doi.org/10.1016/j.jinteco.2018.04.008 (2018).
39 Fontagné, L., Guimbard, H. & Orefice, G. Product-level Trade Elasticities: Worth Weighting For. (CEPII, Paris, 2019).
40 Kuang, Y. & Xiang, H. Who benefits from antidumping and countervailing? an analysis using a computable partial equilibrium model. *Emerging Markets Finance and Trade* **55**, 409-426, doi:10.1080/1540496X.2018.1515735 (2019).
41 WTO. *Tariff Analysis Online facility*, <https://tao.wto.org/welcome.aspx?ReturnUrl=%2f%3fui%3d1&ui=1> (2020).
42 Ministry of Commerce of The People's Republic of China. *Foreign Trade Online Inquiry*, <http://wmsw.mofcom.gov.cn/wmsw/> (2020).
43 Niu, Z., Liu, C., Gunessee, S. & Milner, C. Non-tariff and overall protection: evidence across countries and over time. *Review of World Economics* **154**, 675-703, doi:10.1007/s10290-018-0317-5 (2018).
44 USITC. *Understanding safeguard investigations*, <https://www.usitc.gov/press_room/us_safeguard.htm> (
45 United States Trade Representative (USTR). *China Section 301-Tariff Actions and Exclusion Process*, <https://ustr.gov/issue-areas/enforcement/section-301-investigations/tariff-actions> (2020).
46 USITC. *Import injury investigation-Crystalline silicon photovoltaic cells and modules from China*, <https://www.usitc.gov/investigations/701731/2012/crystalline_silicon_photovoltaic_cells_and_modules/final.htm> (2012).
47 USITC. *Import injury investigatons-Certain crystalline Silicon Photovoltaic Products from China and Taiwan*, <https://www.usitc.gov/investigations/701731/2014/certain_crystalline_silicon_photovoltaic_products/final.htm> (2014).
48 The State Council The people's Republic of China. *Notice on increasing tariff rates on import goods originated in the United States*, <http://www.gov.cn/xinwen/2019-05/13/content_5391208.htm> (2019).
49 European Commission. *Investigations history of proceeding-solar panels (crystalline silicon photovoltaic modules and key components)*, <https://trade.ec.europa.eu/tdi/case_history.cfm?ref=com&init=1895&sta=1&en=20&page=1&number=&prod=module&code=&scountry=all&proceed=all&status=all&measures=all&measure_type=all&search=ok&c_order=name&c_order_dir=Up> (2020).
50 China Daily. *China, EU settle solar panel dispute*, <http://www.chinadaily.com.cn/business/2013-08/06/content_16875082.htm> (2013).
51 China Trade Remedies Information. *India issued final decision on solar cells and modules safeguard measures investigation*, <http://cacs.mofcom.gov.cn/cacscms/articleDetail/ckys?articleId=155122&id=53d8a6e26491a04b

0164b63e2944198d> (2018).
52 EnergyTrend. *India officers suggest tariff imposed on imported photovoltaic modules since 2021*, <https://www.energytrend.cn/news/20190926-76105.html> (2019).
