# Supplementary material for: Breaking down barriers on PV trade will facilitate global carbon mitigation
Source: Nat Commun. 2021 Nov 24;12:6820. doi: 10.1038/s41467-021-26547-7 (PMC8613243; doi:10.1038/s41467-021-26547-7)
Supplement: Supplementary file 3 — Reporting Summary [file 41467_2021_26547_MOESM3_ESM.pdf]

nature portfolio

Corresponding author(s): Mao Xianqiang, Song Peng

Last updated by author(s): Sep 10, 2021

Reporting Summary

Nature Portfolio wishes to improve the reproducibility of the work that we publish. This form provides structure for consistency and transparency in reporting. For further information on Nature Portfolio policies, see our Editorial Policies and the Editorial Policy Checklist.

Statistics

For all statistical analyses, confirm that the following items are present in the figure legend, table legend, main text, or Methods section.

n/a	Confirmed	
☒	☐	The exact sample size (*n*) for each experimental group/condition, given as a discrete number and unit of measurement
☒	☐	A statement on whether measurements were taken from distinct samples or whether the same sample was measured repeatedly
☒	☐	The statistical test(s) used AND whether they are one- or two-sided<br>*Only common tests should be described solely by name; describe more complex techniques in the Methods section.*
☒	☐	A description of all covariates tested
☒	☐	A description of any assumptions or corrections, such as tests of normality and adjustment for multiple comparisons
☒	☐	A full description of the statistical parameters including central tendency (e.g. means) or other basic estimates (e.g. regression coefficient) AND variation (e.g. standard deviation) or associated estimates of uncertainty (e.g. confidence intervals)
☒	☐	For null hypothesis testing, the test statistic (e.g. *F*, *t*, *r*) with confidence intervals, effect sizes, degrees of freedom and *P* value noted<br>*Give P values as exact values whenever suitable.*
☒	☐	For Bayesian analysis, information on the choice of priors and Markov chain Monte Carlo settings
☒	☐	For hierarchical and complex designs, identification of the appropriate level for tests and full reporting of outcomes
☒	☐	Estimates of effect sizes (e.g. Cohen's *d*, Pearson's *r*), indicating how they were calculated

Our web collection on statistics for biologists contains articles on many of the points above.

Software and code

Policy information about availability of computer code

Data collection	There is not custom codes being developed in the study.<br>The version number of GSIM is 'GSIM 6.0'. GSIM models is available from website http://www.i4ide.org/content/wpapers.html.<br>IMS is developed by the Energy and Materials Research Group (EMRG) at Simon Fraser University (SFU). Information about IMS is available from website http://www.sfu.ca/emrg/Our_Research/policy-modelling.html.<br>The version number of TIMES is ' 4.5.834 '. Information about TIMES is available from the website https://www.kanors-emr.org/.
Data analysis	There is not custom codes being developed in the study.<br>The version number of GSIM is 'GSIM 6.0'. GSIM models is available from website http://www.i4ide.org/content/wpapers.html.<br>IMS is developed by the Energy and Materials Research Group (EMRG) at Simon Fraser University (SFU). Information about IMS is available from website http://www.sfu.ca/emrg/Our_Research/policy-modelling.html.<br>The version number of TIMES is ' 4.5.834 '. Information about TIMES is available from the website https://www.kanors-emr.org/.

For manuscripts utilizing custom algorithms or software that are central to the research but not yet described in published literature, software must be made available to editors and reviewers. We strongly encourage code deposition in a community repository (e.g. GitHub). See the Nature Portfolio guidelines for submitting code & software for further information.

Data

Policy information about availability of data

All manuscripts must include a data availability statement. This statement should provide the following information, where applicable:

- Accession codes, unique identifiers, or web links for publicly available datasets
- A description of any restrictions on data availability
- For clinical datasets or third party data, please ensure that the statement adheres to our policy

The processed data for PV trade, embodied carbon flow, future energy mix projections, emission reduction potential and the generated results of trade barrier impacts simulation are provided in Source Data file (Source Data.xlsx). The PV product trade data used in this study are available from multilateral institutional databases, including, the UN Comtrade database (https://comtrade.un.org/), and from databases of various countries/economies' customs and trade departments, e.g., USITC (https://dataweb.usitc.gov/). The life cycle inventory data used in this study are available from Ecoinvent database (https://www.ecoinvent.org/home.html). The data for power generation carbon emission factors calculations for various countries/economies are from various official statistics, e.g., EU countries (https://www.eea.europa.eu/data-and-maps/data/co2-intensity-of-electricity-generation). Other scattered supporting data sources have been linked to the literatures or websites cited in the paper.

Field-specific reporting

Please select the one below that is the best fit for your research. If you are not sure, read the appropriate sections before making your selection.

☐ Life sciences ☐ Behavioural & social sciences ☒ Ecological, evolutionary & environmental sciences

For a reference copy of the document with all sections, see nature.com/documents/nr-reporting-summary-flat.pdf

Ecological, evolutionary & environmental sciences study design

All studies must disclose on these points even when the disclosure is negative.

Study description	This study focuses on carbon emission reduction potential of global PV products trade and the trade barriers on solar cells and modules to carry out analysis. First, trade flow matrix (TFM) is constructed to describe the global PV products trade situation. Second, the carbon embodied in PV products trade are calculated. Third, the net carbon emission reduction potential of PV power generation is estimated and predicted up to 2060 with a bottom-up technology-based model, IMS. Finally, a computable partial equilibrium model, GSIM, is applied to simulate the impacts of trade barriers on PV product trade, and the gains and losses in carbon emissions reduction potential are calculated.
Research sample	Harmonized system (HS) commodity codes are used to retrieve trade data for PV products (HS6 code, silicon-280461, silicon wafer-381800, and solar cells and modules-854140). Fifty-three countries/economies and the "rest of the world" (ROW) are included in the analysis.
Sampling strategy	To form a global PV product trade data set, this study choose the major PV product trade partners, the cumulative share of which exceeded 80% for each product based on the ABRAMS world trade wiki. Harmonized system (HS) commodity codes were used to retrieve trade data for PV products (HS6 code, silicon-280461, silicon wafer-381800, and solar cells and modules-854140). More specific trade data with 8 or 10 digits were obtained from official statistics or customs of various countries/economies. Fifty-three countries/economies and the "rest of the world" (ROW) are included in the TFM, and they are divided into six groups/regions, namely, Oceania, Europe, Southeast Asia (ASEAN plus India and Turkey), Americas, East Asia, and ROW.
Data collection	PV product trade data are extracted by Wang Mudan and Lu Jianhong from multilateral institutional databases, including the UN Comtrade database and databases of customs and trade departments of various countries/economies. The LCA carbon emissions coefficients are obtained by Wang Mudan and Guozhi from the Ecoinvent database. Data for future energy mix projections are drawn from national development plans, energy development outlooks, NDC target commitments, long-term energy strategic plans and decarbonisation pathway research reports of various countries/economies, by Xing Youkai, Wang Mudan and Guo Zhi.
Timing and spatial scale	Date collection started from early 2017 and stopped in late 2020.
Data exclusions	There is no data exclusions in the present study. Because the data we employed are objective statistical trade data and LCA data.
Reproducibility	Following the detailed steps and procedures and using data sets we provided in the method section, reproducibility can be guaranteed.
Randomization	This is not relevant to the present study. Because all the major PV product trade parter and PV application countries/economies are involved in our TFM and IMS/TIMES and GSIM modeling, which is quite deterministic.
Blinding	This is not relevant to the present study. Because the major PV products and their trade parters are involved in our TFM and IMS/TIMES and GSIM modeling, with no need to use blinding strategy.

Did the study involve field work? ☐ Yes ☒ No

Reporting for specific materials, systems and methods

We require information from authors about some types of materials, experimental systems and methods used in many studies. Here, indicate whether each material, system or method listed is relevant to your study. If you are not sure if a list item applies to your research, read the appropriate section before selecting a response.

Materials & experimental systems

n/a	Involved in the study
☒	☐ Antibodies
☒	☐ Eukaryotic cell lines
☒	☐ Palaeontology and archaeology
☒	☐ Animals and other organisms
☒	☐ Human research participants
☒	☐ Clinical data
☒	☐ Dual use research of concern

Methods

n/a	Involved in the study
☒	☐ ChIP-seq
☒	☐ Flow cytometry
☒	☐ MRI-based neuroimaging
